# The Influence of Temperament on Body Temperature Response to Handling in Angus Cattle

**DOI:** 10.3390/ani10010172

**Published:** 2020-01-20

**Authors:** Angela M. Lees, Hannah E. Salvin, Ian. G. Colditz, Caroline Lee

**Affiliations:** 1 CSIRO Agriculture and Food, Animal Behaviour and Welfare, FD McMaster Laboratory, Armidale, NSW 2350, Australia; ian.colditz@csiro.au; 2 School of Environmental and Rural Science, University of New England, Armidale, NSW 2350, Australia; 3 New South Wales Department of Primary Industries, Livestock Industries Centre, Armidale, NSW 2351, Australia; hannah.salvin@dpi.nsw.gov.au

**Keywords:** crush score, exit velocity, flight speed, rectal temperature, stress response, stress-induced hyperthermia

## Abstract

**Simple Summary:**

Understanding animal responses to stressful stimuli is a fundamental aspect to evaluating animal welfare. Stress-induced hyperthermia (SIH) is a term used to describe a short-term increase in body temperature that occurs in response to stressful stimuli. Recently there has been increasing interest in SIH as a physiological measure of psychological stress in livestock species. Previously, studies have suggested that cattle with more excitable temperaments exhibit an increased stress response. This study evaluated the influence of temperament on SIH, during a standardized handling procedure in *Bos taurus* cattle. In this study, body temperature increased, regardless of sex or temperament traits, characterizing SIH. Nevertheless, both flight speed (FS) and crush score (CS) were associated with an elevated rectal temperature (T_REC_) 30 min prior to the handling procedure, and this continued from the start of handling (T0) to 10 min post-handling (T10). The results from this study suggest that temperament may be related to variation in SIH in cattle during handling. Understanding the variation in behavioral and physiological response to stressful events may enable the development of new measures for genetic selection in cattle.

**Abstract:**

Previous studies have indicated that cattle with more excitable temperaments exhibit an increased stress response. The objective of this study was to investigate the relationship between temperament traits, handling, and stress-induced hyperthermia (SIH) in beef cattle. Rectal temperatures (T_REC_, °C) of 60 purebred Angus cattle (30 heifers, 30 steers; 235.2 ± 5.11 kg) were recorded at 20 s intervals from 30 min prior to handling until two hours post handling. All cattle were exposed to a standardized handling procedure consisting of (i) being restrained in a weighing box for 30 s; (ii) being held within the crush for 30 s; and then (iii) being restrained in a head bail for 60 s. Cattle temperaments were evaluated via three traits: (1) agitometer score (AG); (2) crush score (CS); and (3) flight speed (FS) during the handling procedure. Agitometer scores and FS measures were used to describe an AG category (AG_CAT_) and an FS category (FS_CAT_) that were used to classify animals into three temperament categories: 1, calm; 2, intermediate; and 3, temperamental. Pearson’s correlation coefficients were used to evaluate the associations between (i) AG, CS, FS, and T_REC_ 30 min prior to entry into the weighing box (T-30) and then at 1 min intervals between time of entry into the weighing box (T0) until 10 min post-weighing (T10); and (ii) the relationship between AG, CS, and FS. The relationship between T_REC_ and temperament traits over the 2.5 h were modeled by using a first-order autoregressive repeated measures model. Flight speed had strong to moderate associations with T_REC_ at T-30 (r ≥ 0.37; *p* ≤ 0.006) and between T0 and T10 (r ≥ 0.36; *p* ≤ 0.01). There were moderate associations amongst T_REC_ between T0 and T10 and CS (r ≥ 0.31; *p* ≤ 0.01). A weak relationship existed with CS (r = 0.16; *p* = 0.16). There were no associations between AG and T_REC_ at T-30 (r ≥ −0.15; *p* = 0.84) or between T0 and T10 (r ≤ 0.04; *p* ≥ 0.4). Rectal temperature, irrespective of sex and temperament traits, was influenced by time (*p* < 0.0001), and maximum T_REC_ (39.3 ± 0.04 °C) occurred between 4 and 5.7 min after entry into the weighing box. In addition, CS (*p* = 0.007) influenced T_REC_ in these cattle. There were also time × temperament trait × sex interactions with the CS (*p* = 0.0003) and FS_CAT_ (*p* = 0.043) categories; however, time × temperament trait interactions were not statistically significant. Results from this study suggest that cattle with excitable temperaments, as evaluated by FS and CS, have a greater increase in T_REC_. In addition, these results suggest that a relationship exists between basal T_REC_ and FS and CS. Together, these results highlight that temperament, as assessed by FS and CS, influences both basal T_REC_ and the peak temperature recorded following handling but does not influence the magnitude of change in T_REC_ post handling.

## 1. Introduction

In livestock production enterprises, temperament is an important consideration, as individuals that are classified to have more excitable temperaments have been associated with decreased average daily gain [1,2]; reduced carcass quality characteristics [2,3]; and reduced immune function [4,5]. Furthermore, previous studies have suggested that cattle with more excitable temperaments exhibit an increased stress response [6,7,8,9]. As cattle temperament becomes more excitable/temperamental/reactive, their reactivity to human contact and handling procedures can become more aggressive or fearful [10], which may result in a heightened stimulation of catecholamines and glucocorticoids [11,12]. The latter suggests that the functional characteristics of the hypothalamic-pituitary-adrenal axis may vary with animal temperament [8,9]. 

Cattle with a lower rectal temperature (T_REC_, °C) in tropical environments have been reported to have calmer temperaments, greater reproductive efficiency, better growth rate, and a greater resistance to ticks [13,14]. Temperament in cattle is considered to be the individual variation in behavioral responses to stressors or stressful events, including human handling [2,9,10]. Cattle temperament traits may influence how an animal responds to routine handling and husbandry procedures on-farm [10,15]. Furthermore, previous studies have established a relationship between flight speed (FS) and T_REC_ [7,13], and FS has been described to have a moderate to high (≥0.3) heritability [13,16,17,18]. Rectal temperature has been described as moderately heritable in *Bos indicus* [13,14] and *Bos taurus* [19,20] cattle, when evaluated during hot environmental conditions. 

Body temperature in cattle is tightly regulated typically within ±1 °C gradient [21]. Increases in body temperature have been associated with exercise [22], digestion [23], inflammation and activation of the immune system [6,24], hot environmental conditions [25], and psychosocial stressors [26]. Within minutes of exposure to acute physiological stressors, there can be a short-term transient increase in body temperature. This phenomenon is termed stress-induced hyperthermia (SIH) or emotional fever [26], and has been reported in numerous species, including rodents [26], humans [27], sheep [28,29], and, more recently, cattle [30]. 

Previous studies have established that bulls classified as temperamental prior to road transport or a lipopolysaccharide challenge have higher T_REC_ when compared with bulls classified as calm [6,7], suggesting overall that routine handling procedures induce a stress or fear response in cattle [30,31,32]. Understanding how temperament relates to physiological stress responses may further enhance the ability to select animals that have a greater capacity to cope with production systems. 

The objectives of this study were to evaluate the influence of temperament and handling on SIH in *Bos taurus* cattle during a standardized handling procedure. Additionally, within the current study indwelling intra-rectal loggers were used to evaluate the influence of temperament on baseline T_REC_ prior to handling and during a standardized handling procedure in *Bos taurus* cattle. 

## 2. Materials and Methods 

This study was conducted with the approval of the CSIRO McMaster Laboratory Animal Ethics Committee (ARA18-04), in accordance with the guidelines of the Australian National Health and Medical Research Council [33]. Data were collected over a single day during a southern hemisphere autumn (May). The study was undertaken in the New England district of New South Wales, Australia (30.52° S, 151.67° E, 1050 m above mean sea level), at the FD McMaster Research Laboratory. Climatic conditions were obtained between 08:30 h and 14:30 h, to encompass climatic conditions 30 min prior to handling and 2 h post-handling. Climatic data were obtained at 30 min intervals from the Australian Bureau of Meteorology’s (www.bom.gov.au) weather station (ID056238), located at Armidale Airport (30.53° S, 151.62° E, 1079 m above mean sea level). During the handling procedure, average (±SEM) ambient temperature, dew point temperature, relative humidity, and wind speed were 12.3 ± 0.81 °C, 3.1 ± 0.81 °C, 57.2 ± 6.23%, and 2.4 ± 0.12 m/s, respectively.

### 2.1. Animals

Thirty purebred Angus heifers (226.2 ± 5.01 kg) and 30 purebred Angus steers (244.3 ± 8.59 kg), aged between 7 and 10 months, were sourced from the FD McMaster Research Laboratory Angus performance-recorded herd and used in the study. Prior to this study, the FD McMaster Research Laboratory Angus performance-recorded herd was managed as a single herd. Cattle used in this study had previously been yard-weaned over a seven-day period, nine weeks prior to the study. Weaning occurred in a different handling facility to the one used in the current study. During weaning, cattle were fed a mix of lucerne hay (*Medicago sativa*) and lucerne-based pelleted concentrate. After weaning, and throughout the study, cattle were group-housed as a contemporary cohort on grazing pastures (*Phalaris aquatic*, *Dactylis glomerata*, and *Plantago lanceolata*). During the study, cattle were relocated to a paddock adjacent to the cattle-handling facilities and were supplemented with approximately 1.5 kg/head of whole cotton seed (*Gossypium* spp.) daily.

### 2.2. Body Temperature

On day 1, cattle were relocated from a paddock adjacent to the handling facility and intra-rectal data loggers were placed into the rectal cavity of all animals (n = 60). The handling facility was the same facility used for the handling procedure (see below) on day 2. Intra-rectal loggers were prepared and placed in situ using a technique modified from Lea et al. [34]. Briefly, intra-rectal loggers consisted of an iButton (DS1922L, Thermochron iButton Device; Maxim Integrated, San Jose, CA, USA) attached to soft polyethylene piping (180 mm in length × 8 mm in diameter) and fixed in place using heat shrink plastic. The loggers were inserted into the rectum and held in place using veterinary tape (Tensoplast^®^ Vet, BSN Medical Inc., Hamburg, Germany) to attach the exposed end of the logger to the underside of the tail. After intra-rectal loggers were in place, all cattle (n = 60) were returned to grazing pasture adjacent to the handling facility. Data loggers were programed to record T_REC_ at 20 s intervals from 08:00 h on day 2, 1 h prior to the handling procedure until 2 h post handling. 

### 2.3. Handling Procedure

At 08:00 h, on day 2, cattle were relocated from the adjoining paddock and marshalled into holding yards within the cattle handling facility, used on day 1. Cattle were given 30 min to settle down and return to basal activity prior to marshalling into a single-file race. Cattle were then exposed to a standardized handling procedure, typical of routine husbandry, consisting of three components: (1) cattle being confined in a single animal-weighing box (Ramage Engineering, Guyra, Australia, fitted with TRUTEST weighing cells and the XR3000 indicator panel) with solid sides, for 30 s (stage 1); then (2) being confined within a crush (MK5, Ramage Engineering, Guyra, Australia) for 30 s (stage 2); and then (3) being restrained in a head bail for 60 s (stage 3). The duration of the handling procedure was 2.3 min for each animal. A similar procedure utilized by Lee et al. [35], involving restraint in a head bail, was shown to elicit an acute stress response as quantified by increased cortisol and beta-endorphin concentrations. All cattle were subjected to the handling procedure, which occurred over approximately 3 h on day 2. 

### 2.4. Temperament Assessments

During the handling procedure on day 2, cattle temperaments were evaluated by utilizing three methodologies: (1) agitometer score (AG), (2) crush score (CS), and (3) flight speed (FS). Agitometer scores were determined via a purposely built agitometer attached to the weigh box via a metal bracket, modified from Blache and Ferguson [36]. The agitometer was designed and developed by Blache and Ferguson [36] and is used to measure the vibrations over a 30 s period, providing a numerical value of cattle movement within the weigh box, during stage 1 of the standardized handling procedure, as described above in Section 2.3. The agitometer was calibrated with a specifically engineered unit that mimicked the action of animals through four ‘feet’ via spring-loaded solenoids, as described by Blache and Ferguson [36]. 

Crush scores were evaluated by a single trained observer over the entire 30 s period, whilst the cattle remained unrestrained within the crush, during stage 2 of the standardized handling procedure, as described above, in Section 2.3. Crush scores were evaluated by using a 5-point subjective scale scoring system, as described in Table 1.

Flight speeds were determined for all cattle on exit from the crush. Flight times were recorded for the time taken for cattle to traverse through two pairs of infrared sensors 1.8 m apart (FarmTek Electronic Timers, Wylie, TX, USA). These data were used to calculate FS (m/s) as described by Burdick et al. [39] (FS = m/time, s), and flight speed data were then used for statistical analysis. 

### 2.5. Statistical Analysis

Seven data loggers failed to download data, and six data loggers provided data that were deemed erroneous, i.e., T_REC_ <20 °C. Additionally, two animals physically displaced the infrared sensors that were recording flight time; thus, data from a total of 15 animals were excluded from analysis. Data analyzed incorporated information from 22 heifers and 23 steers. All data exploration and statistical analyses were conducted in R [40]. 

Rectal temperatures were aligned for each animal, so that time zero (T0) occurred at entry into the weighing box. Individual T_REC_ data sets were generated to encompass a 2.5 h period consisting of data from 30 min prior to entry into the weigh box to 2 h post-entry into the weigh box. Baseline T_REC_ was considered as time −30 min, i.e., the first measurement of T_REC_ 30 min prior to entry into the weigh box. Change in T_REC_ was considered the difference in T_REC_ for each 20 s time point for comparison to baseline T_REC_ (T-30). 

Agitometer scores were used to categorize animals into three temperament categories (AG_CAT_): 1, calm, ≤33 (n = 16; ♀ = 6, ♂ = 10); 2, intermediate, 45 ≤ 82 (n = 15; ♀ = 10, ♂ = 5); and 3, temperamental, ≥86 (n = 14; ♀ = 6, ♂ = 8). Similarly, FS were then classified into three temperament categories (FS_CAT_): 1, calm, ≤1.6 m/s (n = 13; ♀ = 2, ♂ = 11); 2, intermediate, 1.7 m/s ≤2.5 m/s (n = 23; ♀ = 14, ♂ = 9); and 3, temperamental, ≥2.6 m/s (n = 9; ♀ = 6, ♂ = 3). Agitometer and flight speed categories were developed by applying a percentile ranking technique, based on the data generated within this study [12,39,41]. Crush scores of 5 were not observed within the current study; thus, CS were grouped into 4 categories, as previously described: (1) calm (n = 14, ♀ = 4, ♂ = 10); (2) slightly restless (n = 16, ♀ = 7, ♂ = 9); (3) restless (n = 11; ♀ = 9, ♂ = 2); and (4) nervous (n = 4; ♀ = 3, ♂ = 1).

Initially, Pearson’s correlation coefficients were used to evaluate the relationship between temperament measures. Then, the influence of sex on temperament traits were evaluated by Welch’s two-sample t-test. Pearson’s correlation coefficients were then used to evaluate the relationship between temperament traits (AG, CS, and FS) and T_REC_ at T-30 and at one-minute intervals between T0 to T10. 

Due to the proximate relationship and highly correlated nature of T_REC_ measures, the relationship between T_REC_, sex, and temperament traits (CS, AG_CAT_, and FS_CAT_) were modeled by using a first-order autoregressive repeated measures model, within the “nlme” package [42]. The influence of temperament traits on T_REC_ were investigated individually, as previous studies have established correlations between temperament traits. Each model incorporated time (each 20 s interval); a temperament trait (CS, AG_CAT_, or FS_CAT_); sex; time × sex; time × temperament trait (CS, AG_CAT_, or FS_CAT_); and time × temperament trait (CS, AG_CAT_, or FS_CAT_) × sex as fixed effects. Animal identification was incorporated as a random effect in all models. Initially live weight was evaluated as a covariate within all models, but did not contribute significance (*p* ≥ 0.05); thus, live weight was excluded from the final models. 

Statistical significance was set at *p* < 0.05, and tendencies were determined if *p* ≥ 0.05 and ≤ 0.10. The strength of the Pearson’s correlation coefficients was identified as weak (r ≤ 0.30), moderate (r = 0.30 ≤ 0.50), and strong (r ≥ 0.50), as described by Cohen [43]. 

## 3. Results

**Figure 1:**
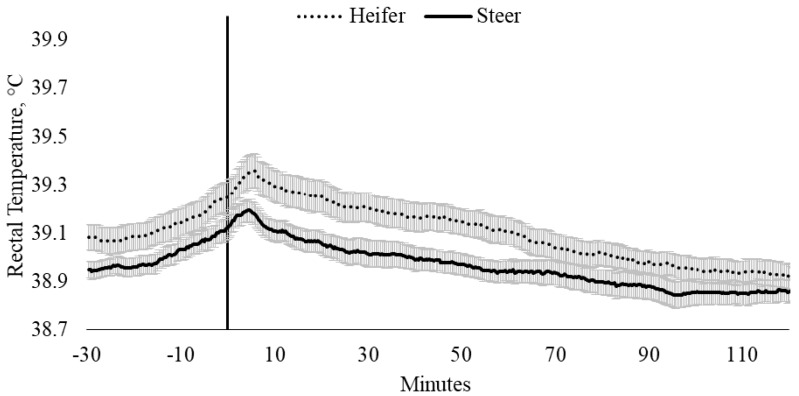
Twenty-seconds mean (±SEM) rectal temperature (T_REC_, °C) of heifers (dotted line) and steers (solid line), 30 min prior to 2 h post-handling, also showing the time from weighing box (solid black vertical line).

**Figure 2:**
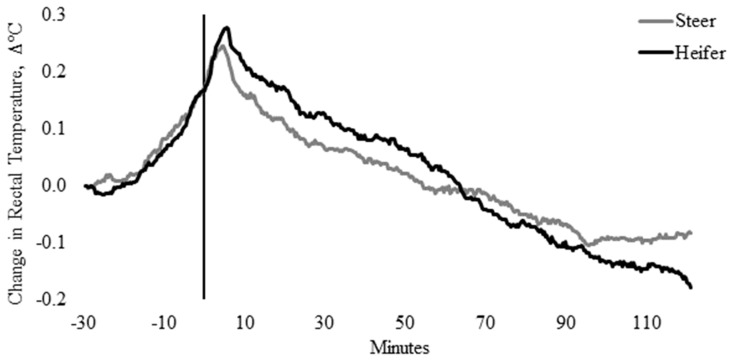
Change in twenty-seconds rectal temperature (±SEM) from baseline rectal temperature (T_REC_, °C) for heifers (solid black line) and steers (solid gray line), 30 min prior to 2 h post-handling, also showing the time from weighing box (solid black vertical line).

Rectal temperature, irrespective of sex and temperament traits, was influenced by time (*p* < 0.0001). Pooled T_REC_ data indicated that maximum T_REC_ (39.3 ± 0.04 °C) occurred 4 min after entry into the weighing box (T0; *p* = 0.008) and remained elevated until 5.7 min. There were no differences in the T_REC_ between heifers (39.4 ± 0.07 °C) and steers (39.2 ± 0.05 °C; Figure 1), nor were there differences in the change in T_REC_ between heifers and steers from baseline T_REC_ (*p* = 0.596; Figure 2). Change in T_REC_ peaked between 3.3 and 5.0 min and at 5.7 min for steers (0.24 °C) and heifers (0.28 °C), respectively, although the change in T_REC_ of heifers was 0.27 °C between 4.3 and 5.3 min (Figure 2). Post-handling, T_REC_ showed a steady rate of decline (−0.01 °C) between 6.0 and 90.4 min, stabilizing at 38.9 ± 0.03 °C (Figure 1 and Figure 2). After 90.4 min, T_REC_ remained within a temperature range between 38.88 ± 0.03 °C and 38.92 ± 0.04 °C, until data loggers were removed, 120 min post-handling (Figure 1 and Figure 2).

There were no relationships identified between FS and CS (r = 0.09; *p* = 0.28) or FS and AG (r = 0.05; *p* = 0.36). Heifers had greater CS (*p* = 0.017) and FS (*p* = 0.008) when compared to steers, however, there were no sex differences in AG (*p* = 0.75; Table 2).

There were strong to moderate associations between T-30 T_REC_ and FS (r = 0.51; *p* = 0.0002). There was a weak relationship between CS and T-30 T_REC_ (r = 0.16; *p* = 0.16). However, there were no relationships with T-30 T_REC_ and AG (r = −0.15; *p* = 0.84).

There were strong to moderate associations between FS (r ≥ 0.50; *p* ≤ 0.0001) and T_REC_ between T0 and T10 (Table 3). Similarly, there were moderate associations amongst T_REC_ between T0 and T10 and CS (r ≥ 0.31; *p* ≤ 0.01; Table 3). There were no relationships between T_REC_ and AG (r ≤ 0.03; *p* ≥ 0.42) at any time point between T0 and T10, highlighting that the correlations are not changing over this period (Table 3). 

**Figure 3:**
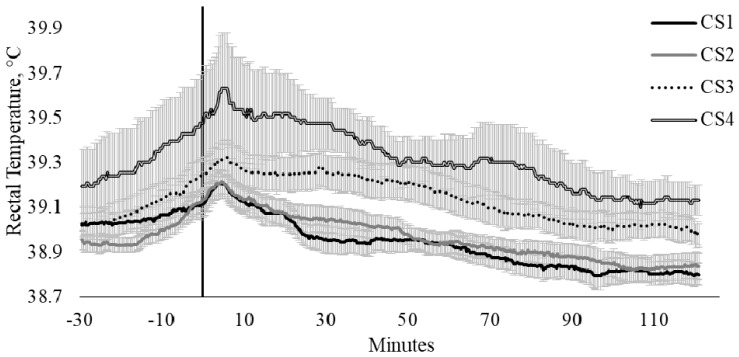
Twenty-seconds mean (±SEM) rectal temperature (T_REC_, °C) of cattle crush scores (CS) of 1 (CS1; n = 14; ♀ = 4, ♂ = 10); 2 (CS2; n = 16; ♀ = 7, ♂ = 9); 3 (CS3; n = 11; ♀ = 9, ♂ = 2); and 4 (CS4; n = 4; ♀ = 3, ♂ = 1).

Crush score had a strong relationship with T_REC_ (CS, *p* = 0.007). There were limited differences between the T_REC_ of CS scores 1 (calm) and 2 (slightly restless), where the minimum and maximum temperature differences were −0.10 and 0.11 °C, respectively (Figure 3); however, as the CS category increased, there was an increase in the difference in T_REC_ (min, 0.01 °C; max, 0.52 °C; Figure 3). The influence of CS on T_REC_ were particularly evident between cattle scored as category 1 (calm; n = 14) when compared with cattle scored as category 4 (nervous, n = 4), where cattle that were scored in category 1 exhibited a 0.18 °C increase in T_REC_, whilst cattle scored within category 4 had a T_REC_ increase of 0.44 °C (Figure 3). 

**Figure 4:**
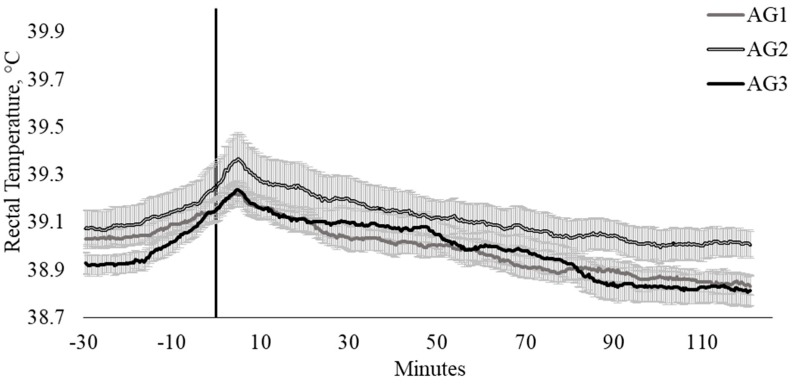
Twenty-seconds mean (±SEM) rectal temperature (T_REC_, °C) of cattle with agitometer scores ≤ 33 (AG1; n = 16; ♀ = 6, ♂ = 10); 45 ≤ 82 (AG2; n = 15; ♀ = 10, ♂ = 5); and ≥ 86 (AG3; n = 14; ♀ = 6, ♂ = 8).

Agitometer category had a tendency (*p* = 0.087) to influence T_REC_ (Figure 4). Interestingly, cattle classified as intermediate temperaments within the AG_CAT_ (AG scores, 45 ≤ 82) were inclined to have higher T_REC_ than cattle with lower (≤ 33) and higher (≥ 86) AG scores (Figure 4). 

**Figure 5:**
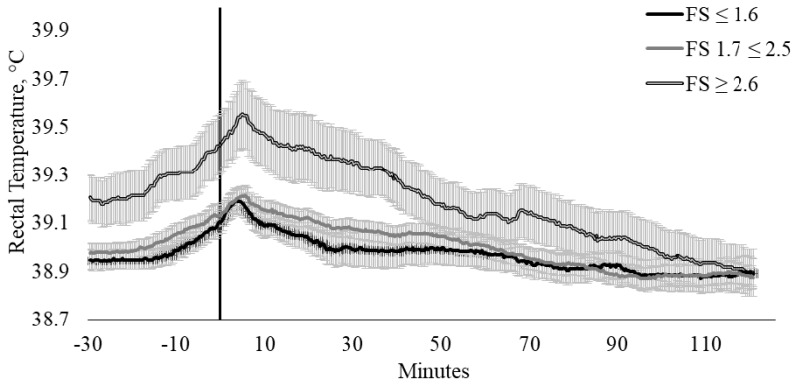
Twenty-seconds mean (±SEM) rectal temperature (T_REC_, °C) of cattle with flight speeds of ≤ 1.6 m/s (FS ≤ 1.6; n = 13; ♀ = 2, ♂ = 11); 1.7 ≤ 2.5 m/s (FS 1.7 ≤ 2.5; n = 23; ♀ = 14, ♂ = 9); and ≥ 2.6 m/s (FS ≥ 2.6 m/s; n = 9; ♀ = 6, ♂ = 3).

Similarly, FS_CAT_ had a tendency to influence T_REC_ (*p* = 0.080) in these cattle (Figure 5). Cattle within FS_CAT_ 3 (FS ≥ 2.6 m/s) generally had higher T_REC_ when compared with FS_CAT_ 1 (FS ≤ 1.6 m/s) and 2 (FS 1.7 ≥ 2.5 m/s). The influence of FS on T_REC_ appears to exist between FS_CAT_ 1 and 2 in comparison to FS_CAT_ 3, where cattle that were within FS_CAT_ 1 and 2 exhibited a 0.22 and 0.24 °C increase in T_REC_, whilst cattle within category 3 had a T_REC_ increase of 0.36 °C. 

**Figure 6:**
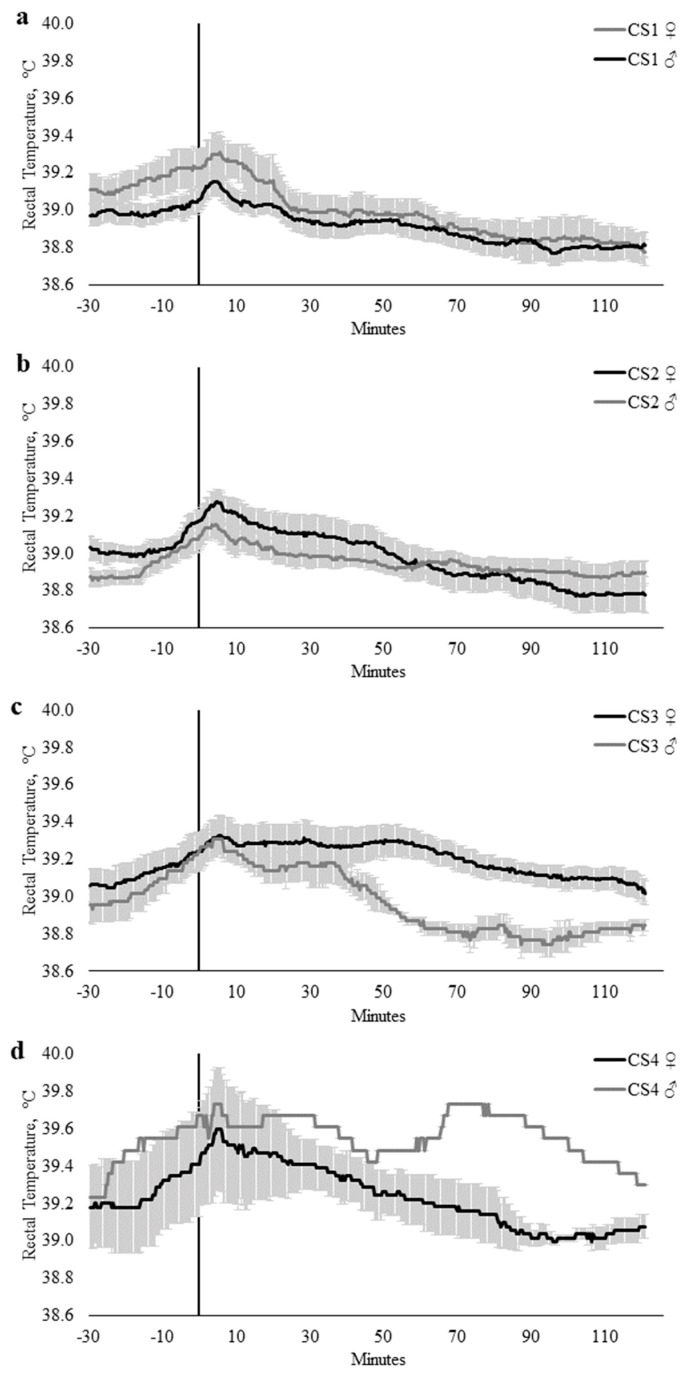
Twenty-seconds mean (±SEM) rectal temperature (T_REC_, °C) of heifers (gray line) and steers (black line) with crush scores of (**a**) 1 (CS1; n = 14; ♀ = 4, ♂ = 10); (**b**) 2 (CS2; n = 16; ♀ = 7, ♂ = 9); (**c**) 3 (CS3; n = 11; ♀ = 9, ♂ = 2); and (**d**) 4 (CS4; n = 4; ♀ = 3, ♂ = 1).

**Figure 7:**
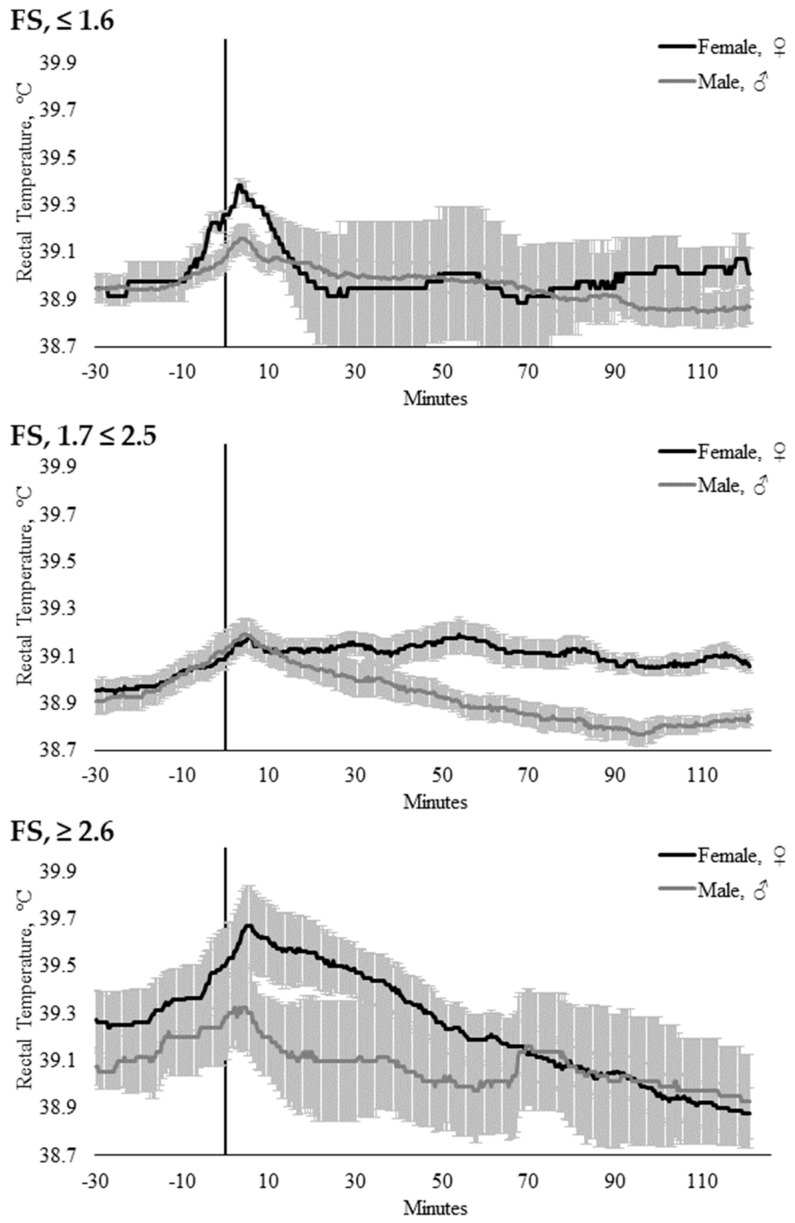
Twenty-seconds mean (±SEM) rectal temperature (T_REC_, °C) of heifers (gray line) and steers (black line) with flight speeds of FS ≤ 1.6 (n = 13; ♀ = 2, ♂ = 11); FS 1.7 ≤ 2.5 (n = 23; ♀ = 14, ♂ = 9); and FS ≥ 2.6 (n = 9; ♀ = 6, ♂ = 3).

Sex did not influence T_REC_ when modeled with AG_CAT_ (*p* = 0.178), CS (*p* = 0.306), or FS_CAT_ (*p* = 0.126). There were no interactions between time × temperament trait, time × sex, or temperament trait × sex within the AG_CAT_, FS_CAT_, or CS models (Table 3). However, there were significant time × temperament trait × sex interactions within the CS (*p* = 0.0003; Figure 6) and FS_CAT_ (*p* = 0.043; Figure 7) models (Table 4). 

## 4. Discussion

Handling cattle can be considered a stressor eliciting a series of responses to stressful stimuli, including SIH [28,29,30,32,35]. Within the current study, maximum T_REC_ (39.3 ± 0.04 °C) occurred between 4 and 5.7 min after entry into the weighing box (T0), and then exhibited a steady rate of decline until 90.4 min, stabilizing at 38.9 ± 0.03 °C. The handling procedure had a total duration of 2.3 min; thus, T_REC_ peaked 2 to 3.7 min after the conclusion of the handling procedure. These results indicate that the change in T_REC_ from the onset of handling was characterizing SIH in these cattle via the short-term increase in T_REC_ that occurred within minutes of the handling procedure commencing [26,44,45]. This is supported by the findings of Sanger et al. [28], concluding that shearing sheep resulted in SIH between 4 and 14 min post-shearing. Therefore, the results presented here provide further evidence that handling is perceived as a stressful event by cattle, which is associated with the induction of a series of responses, including SIH [28,29,30,32,35]. 

There have been numerous studies investigating the impact of various stressors on body-temperature responses in cattle [6,7,22,28,30,46,47]; however, studies investigating the influence of temperament on body temperature are limited. In the current study, AG and FS were used to classify cattle into temperament categories within this study, in line with previous studies [12,41]. The AG device registers vibrations from movement and sound, thus differing from the mechanical movement device which has been previously used in cattle [48,49]. Although the use of this evaluation method was novel for cattle, the AG has been successfully used in sheep studies [36,50,51]. Within the current study, there was no relationship between FS and AG. Similarly, Blache and Ferguson [36] reported a limited relationship (r < 0.01) between flight time and AG in sheep. Within the current study, there were weak to moderate relationships between AG and CS. However, it is not clear what aspects of temperament AG may be evaluating, indicating that FS and AG describe different components of temperament [36]. Further studies investigating the use of AG in cattle, and sheep, and its relationship with other measures of temperament, are required, in order to validate its use in cattle. 

Within the current study, CS appeared to have the greatest influence on T_REC_. When evaluating the impact of CS on T_REC_, there was a clear divergence in T_REC_ responses of cattle across the CS categories. This is particularly evident when comparing the T_REC_ of cattle that were scored within category 1 (calm; n = 14) and category 4 (nervous, n = 4). Similarly, Burdick et al. [7], established that, 30 min following the commencement of transport, temperamental bulls had a greater maximal T_REC_. However, the magnitude of T_REC_ increase was comparable between temperament classes [7], which were also observed within the current study. Whilst somewhat preliminary in nature, the results from the current study suggest that peak body temperature following handling is higher in cattle that are identified to have more excitable temperaments, even though the magnitude of temperature increase is comparable across temperament classes. Overall, the findings of the current study and those of Burdick et al. [7] suggest that temperament classifications may allow for the early identification of cattle with increases in stress responsiveness, manifesting as elevated SIH. However, further studies with greater cattle numbers across temperament scores are required to definitively establish the relationship that exists between temperament traits and SIH.

Recently, there has been some conjecture regarding what aspects of temperament FS and CS are evaluating [12,30,52,53,54]. MacKay et al. [54] suggested that CS evaluate cattle’s response to handling, whereas FS may provide a measure of fearfulness. Moreover, King et al. [12] suggested that FS and CS were related to the stress responsiveness of cattle. However, a recent study by Lee et al. [30] concluded that pharmacologically induced anxiety did not influence FS or CS in cattle, suggesting that these temperament traits do not measure aspects related to anxiety. As such, FS may be a better methodology to evaluate cattle temperament, as its evaluation is objective, whereas subjective evaluations such as CS may allow for human error or bias to influence temperament evaluations [41]. Moreover, FS can be evaluated without restricting animal movement or inducing fear responses from being confined within a crush [37,39] or from social isolation [55]. 

Within the current study, heifers had greater CS and FS when compared with the steers, which is consistent with previous studies [16,38,56], although some studies have suggested that FS does not differ between bulls and heifers [39,57]. Regardless, sex did not influence T_REC_ in these cattle, regardless of temperament traits, nor were there time × sex or temperament trait × sex influences (Table 4). Furthermore, within this study, a relationship between FS and CS was not observed, whilst previous studies had identified weak relationships (r ≤ 0.3) between FS and CS [12,18,41,58,59,60]. Therefore, the results presented here provide further evidence that FS and CS are potentially evaluating different aspects of cattle behavior that contribute to temperament [2,18]. 

In this study, there were strong to moderate associations of T_REC_ between T0 and T10 with FS. Burdick et al. [7] also established a strong relationship between FS and maximum T_REC,_ and FS tended to be correlated with minimum T_REC_. The current study also established moderate relationships between CS and T_REC_ between T0 and T10. Furthermore, there was a strong to moderate association with T-30 T_REC_ and FS identified within this study. Burdick et al. [7] established that temperamental bulls had higher T_REC_ when compared with bulls that were classified as calm and intermediate temperaments (*p* < 0.05) prior to transportation. Similarly, Burdick et al. [6] concluded that temperament influenced T_REC_ in Brahman bulls, where bulls classified as temperamental had higher T_REC_ (*p* < 0.001) prior to a lipopolysaccharide challenge. The relationship between T-30 T_REC_ and FS and CS within this study suggests that these temperament traits may have systemic influence on body temperature regulation, i.e., that cattle with faster FS and higher CS have higher body temperature when not being handled or exposed to stressful stimuli. This may provide a biological rationalization for the elevated T_REC_ in bulls classified as temperamental prior to transport and a lipopolysaccharide challenge as described by Burdick et al. [7] and Burdick et al. [6]. Burrow [13] reported a moderate negative phenotypic correlation (r = −0.24) between flight time and T_REC_, suggesting that cattle with lower T_REC_ have higher flight time. However, the correlations described by Burrow [13] were collected in accordance with the Iberia heat tolerance test, where T_REC_ is collected after cattle have been exposed to ambient temperatures ≥ 30 °C, without access to shade, for a minimum of 3 h [61]; thus, T_REC_ may have been confounded by heat load [25,61,62]. These findings suggest that further investigations are required to determine the relationship between temperament traits and the regulation of body temperature under conditions classified as non-stressful. This is especially pertinent given that heritability estimates for FS and flight time have been described as moderate to high (≥0.3) [13,16,17,18]. Investigating the influence of temperament traits on body temperature may be of importance in the changing global environment [63], as the results from this study suggest that cattle with more excitable temperaments may have higher basal T_REC_, which may indicate that these animals may have an increased susceptibility to heat load. However, further studies are required to quantify the relationship between temperament and body temperature, and the influence that this may have on an animals’ susceptibility to heat load. 

Interestingly, within the current study, T_REC_ 120 min post-handling was 0.13 °C lower than 30 min prior to the handling procedure. Intra-rectal loggers were inserted the day prior to handling, to negate handling and data logger insertion influences on T_REC_. However, these cattle were kept in grazing pastures adjacent to the handling facility and required mustering from the paddock prior to the commencement of the handling procedure. Beatty et al. [47] highlighted that transport, shearing and sorting elevated the core body temperature of sheep. Similarly, Mader et al. [22] reported that moving feedlot cattle from home pens to handling facilities (between 150 and 900 m) increased tympanic temperature between 0.3 and 0.8 °C, within 30 min of cattle movement. Therefore, it is likely that basal T_REC_ described within this study does not provide a true estimate of basal T_REC_, as the T_REC_ presented here may be confounded by relocation and handling events prior to the commencement of the handling procedure. Thus, it is anticipated that this accounts for the elevated T_REC_ prior to the commencement of the handling procedure. Additionally, it is important to consider that body temperature exhibits a circadian rhythm [64,65,66], which may influence the magnitude of the increase in body temperature within SIH studies. Future studies investigating SIH should consider evaluating the underlying circadian rhythm of body temperature prior to exposure to stressful stimuli. Furthermore, given that body temperature is highly regulated [21], understanding the circadian rhythm would allow for SIH to be evaluated by expressing the change in body temperature from a quantified underlying rhythmic temperature. This may provide a more concise estimate of the impact of stressors on body temperature and the time required for body temperature to return to a baseline temperature after exposure to stressors. Quantifying the influence of stressors may provide a more consistent estimate of the impact of stressors on body temperature, thus providing a more reliable physiological evaluation of the impact of stressors on cattle. Furthermore, evaluating temperament is an important consideration for all facets of cattle production, as cattle with excitable/poor/reactive temperaments have been associated with decreased average daily gain [1,2], greater hypothalamic–pituitary–adrenal axis response to handling [9], and reduced carcass quality [2]. Thus, cattle that are considered to have temperamental or excitable temperaments may have reduced welfare regardless of the management procedure being conducted.

## 5. Conclusions

The variations in T_REC_ observed within this study suggest that the standardized handling procedure elicited a temperature response that was characteristic of SIH associated with psychological stress. Basal T_REC_ differed between temperament classes within FS and CS traits, suggesting that there is an underlying influence of temperament traits on body temperature. Cattle with excitable temperaments, as evaluated by FS and CS, had higher T_REC_ following handling; however, the magnitude of the temperature increase was comparable across temperament classes. Further studies are required to definitively quantify the relationship between temperament traits, body temperature, and SIH. Additionally, these results suggest that the impact of relocation, handling and temperament need to be considered when evaluating body temperature in cattle. Overall, these results further highlight the importance of selecting animals for calm temperaments, as cattle with excitable temperaments may exhibit a greater stress response during routine handling procedures.

## Figures and Tables

**Table 1:** Cattle crush scoring descriptions ^1^.

Score	Classification	Descriptor
1	Calm	standing still, head mostly still, and slow, calm movements
2	Slightly restless	looking around more quickly, moving feet, and shifting weight
3	Restless	moving backward and forward, and some slight movement of crush
4	Nervous	continuous vigorous movement backward and forward, snorting, and some movement of crush
5	Very Nervous	violent movements, rearing, and attempting to jump out

^1^ Modified from Lee et al. [30], Cafe et al. [2], Grandin [37], and Voisinet et al. [38].

**Table 2:** Mean (±SEM) agitometer score (AG), crush score (CS) s, and flight speed (FS, m/s) for heifers, steers, and pooled data.

Item	Heifers	Steers	Pooled
AG	64.9 ± 5.11	60.9 ± 6.80	62.9 ± 6.04
CS	2.5 ± 0.14 ^a^	1.8 ± 0.12 ^b^	2.1 ± 0.14
FS	2.3 ± 0.09 ^a^	1.8 ± 0.07 ^b^	2.1 ± 0.09

^a–b^ Different superscripts within rows indicates a significant difference between sexes (*p* < 0.01).

**Table 3:** Pearson’s correlation coefficients between rectal temperature and agitometer score (AG ^1^), flight speed (FS ^1^, m/s), and crush score (CS) between the time of weighing (T0) and 10 min post-weighing (T10).

Item	T0	T1	T2	T3	T4	T5	T6	T7	T8	T9	T10
AG	−0.001	−0.004	−0.01	0.001	0.01	0.03	0.01	0.01	0.003	0.003	0.004
FS	0.53 **	0.52 **	0.53 **	0.52 **	0.53 **	0.56 **	0.58 **	0.57 **	0.59 **	0.59 **	0.58 **
CS ^2^	0.34 *	0.34 *	0.32 *	0.31 *	0.34 *	0.34 *	0.37 *	0.35 *	0.36 *	0.37 *	0.37 *

^1^ Non-categorized agitometer scores and flight speed. ^2^ Crush score, categories: (1) calm (n = 14); (2) slightly restless (n = 16); (3) restless (n = 11); and (4) nervous (n = 4). * denotes *p* ≤ 0.01; ** denotes *p* < 0.0001.

**Table 4:** Interactions (*p*-values) for agitometer score category (AG_CAT_), crush score (CS), and flight speed category (FS_CAT_) models.

Item	AG_CAT_^1^	CS_30_^2^	FS_CAT_^3^
Time	<0.0001	<0.0001	<0.0001
Temperament trait	0.087	0.007	0.080
Sex	0.178	0.306	0.126
Time × sex	0.712	0.633	0.676
Time × temperament trait	0.667	0.112	0.243
Temperament trait × sex	0.915	0.190	0.961
Time × temperament trait × sex	0.618	0.0003	0.043

^1^ Agitometer score, categories: (1) ≤ 33 (n = 16); (2) 45 ≤ 82 (n = 15); and (3) ≥ 86 (n = 14). ^2^ Crush score, categories: (1) calm (n = 14); (2) slightly restless (n = 16); (3) restless (n = 11); and (4) nervous (n = 4). ^3^ Flight speed, categories: (1) ≤ 1.6 m/s (n = 13); (2) 1.7 m/s ≤ 2.5 m/s (n = 23); and (3) ≥ 2.6 m/s (n = 9).
